# Composition and Dynamics of Plant- and Soil-Associated Microbial Communities in Forest and Agricultural Ecosystems

**DOI:** 10.3390/microorganisms11071782

**Published:** 2023-07-10

**Authors:** Tim Dumonceaux

**Affiliations:** 1 Agriculture and Agri-Food Canada Saskatoon Research and Development Centre, Saskatoon, SK S7N 0X2, Canada; tim.dumonceaux@agr.gc.ca; Tel.: +1-306-491-1768; 2 Department of Veterinary Microbiology, University of Saskatchewan, Saskatoon, SK S7N 5B4, Canada

Peter Kropotkin (1842–1921) is well known as an anarchist intellectual, an amiable mass of contradictions who loved humanity and was highly regarded in academic and intellectual circles, yet also penned “fiery peans to violence” in *Le Révolté*, the anarchist journal he established with Elisée Reclus in the 1870s [1]. Less well known, but at least as important, is the fact that Kropotkin was a naturalist who commented not only on revolutionary reforms to society, but also on evolutionary processes in light of contemporary thoughts on the nature of competition as a driving force for natural selection in the decades following the publication of evolutionary theory in 1859. In response to Huxley’s highly influential ideas that emphasized merciless and bloody conflict among organisms as the primary factor driving evolutionary change, Kropotkin published a series of essays in the *nineteenth century*, ultimately collected into a book, *Mutual Aid: A Factor in Evolution* [2]. In it, Kropotkin pointed out that two modes of the struggle for existence had been proposed: “direct” competition among organisms in a context of limited resources, resulting in conflict, and what Darwin referred to as a “metaphorical” struggle of groups of organisms against dynamic and challenging environmental conditions—the plant at the edge of a desert struggling against drought. The first chapters of Kropotkin’s book provide numerous examples he observed during five years in Siberia of organisms expressing a mutualistic, cooperative mode of interaction in a struggle for reproductive success in a harsh environment. He argued that this mode of struggle—where organisms work together in social cohesion to achieve collective aims and protect one another from the worst cruelties of the external environment—represented, in fact, the more important factor in evolutionary change:

*The first thing which strikes us as soon as we begin studying the struggle for existence under both its aspects—direct and metaphorical—is the abundance of facts of mutual aid, not only for rearing progeny, as recognized by most evolutionists, but also for the safety of the individual, and for providing it with the necessary food. With many large divisions of the animal kingdom mutual aid is the rule. Mutual aid is met with even amidst the lowest animals, and we must be prepared to learn some day, from the students of the microscopical pond-life, facts of unconscious mutual support, even from the life of micro-organisms.*

For all of the difficulties with Kropotkin’s political thought, his biological work, while imperfect, was roughly correct. Kropotkin was, as Gould notes, not a “crackpot” [3]. In particular, his views, cited above, on the modes of interaction within the microbial world have proven to be prescient. Studies of host–microbe, microbe–microbe, and microbe–environment interactions are revealing the range and nature of these interactions, which provide important conceptual lessons for our understanding of microbial ecosystems, and point to management strategies that exploit the natural interactions of the microbial and multicellular components of these ecosystems to optimize their health and productivity.

Using examples from natural and managed forest ecosystems, managed agricultural ecosystems, and mixed forestry–agricultural ecosystems, the collection of manuscripts represented in this two-volume set provides examples of these different modes of interaction and offers ideas on exploiting these interactions to achieve productivity aims. In addition, these works highlight some of the limitations of our modern methods of studying these ecosystems, which must provide accurate, complete, reproducible, and unbiased views of microbial ecosystems so that accurate inferences may be drawn from the application of these powerful DNA sequencing-based methods.

One of the most well-known examples of beneficial inter-Kingdom interactions is the plant–fungal mycorrhizal symbiosis. Yokoya et al. [4] assessed the diversity of cultured fungi that form mycorrhizal associations with African orchids (*Cynokis* spp.) in Madagascar, a geographic area with a high density and diversity of species. Critically, they examined the characteristics of the soil ecological niche harboring these fungi and determined that the knowledge of soil characteristics, including N and P, is essential for the successful establishment of mycorrhizal associations and plant health. Plant–mycorrhizal associations determine the success of the re-introduction of endangered orchid species, as exemplified by the work of Sarasan et al. [5] examining the very rare yellow early march-orchid (*Dactylorhiza incarnata* subsp. ochroleuca). This plant is native to the UK, and efforts to sustain this species were supported by the isolation of a mycorrhizal fungus (Family Tulasnellaceae) from a related orchid species. This fungal symbiont facilitated seed germination, and the colonized seedlings were able to establish in the sites into which they were re-introduced. Soil analysis at the new site allowed the establishment of the appropriate micronutrient (P) environment similar to that from which wild plants were obtained, which facilitated the establishment of the re-introduced orchid plants.

Soil is the matrix within which these ecological interactions occur, and it represents the physical interface between the plant roots and the microbial ecosystems that can determine plant health or disease. However, soil is highly heterogeneous, and this feature is an important determinant of the root-associated fungi found in temperate forests. Khokon et al. [6] examined the fungal species associated with plant roots in geographically diverse experimental forest plots to determine the composition of fungi along a vertical axis. Saprotrophic and symbiotrophic fungi are associated with different layers, although the latter are numerically dominant. Certain taxa were considered to be a phylogenetic “signature” for different soil layers, but at a taxonomic level (Order) that provides flexibility in habitat colonization of plant roots by these fungal species. Zverev et al. [7] explored the relationship between plant species diversity and bacterial taxonomic diversity in the rhizosphere. Using a plant taxonomic marker amplified from root biomass in combination with bacterial 16S rRNA genes, they determined that alpha diversity metrics (referring to the richness and evenness of species distribution) are not related between plant taxa and rhizosphere bacteria, but beta diversity measures (community compositions) are strongly correlated. Terrestrial soil is not the only matrix that provides an ecological interface between plants and microbial taxa. Vascular epiphytes, non-parasitic plants that grow and are dependent on other plants, form their own “suspended” soil that is not in contact with the terrestrial soil. Eskov et al. [8] examined such soils, providing the first glimpse of these unique microbial communities. Remarkably, the suspended soils house a treasure trove of microbial diversity, primarily due to the accumulation of organic matter that was protected from insect (termite) degradation as a result of its location off of the ground.

Forest soil is continuously re-generated due to the decomposition activity of soil microbial communities, which also cycle nutrients and support plant growth and productivity. The decomposition of mulched plant litter from *Cinnamomum migao*, an evergreen native to China, was examined in a year-long experiment [9]. Urease and invertase activities were increased by mulching, and microbial taxa were identified as parts of communities that differed in their composition and network structures according to soil organic carbon and catalase activities. These observations have implications for the management of this important forest species in China. Species diversity is important for ecosystem sustainability. Liu et al. [10] examined the effects of utilizing mixed tree species (*Pinus sylvestris* and *Morus alba*) for soil erosion mitigation on microbial biodiversity. Litter decomposition by soil microbial species was used as a metric for exploring the ecological relationships among introduced tree species, since forest litter is an important player in nutrient cycling in these ecosystems. The results showed that mixed stands display significantly higher litter decomposition rates and have a higher diversity of litter-associated microbial communities. These observations suggest that the use of multiple tree species offers improvements over monocultures for soil management due to their interspecific relationships, mediated through the litter decomposing microbial communities.

The examination of soil bacterial and fungal microbiota and their interactions with each other and their plant hosts can inform agricultural and forestry management practices. For example, agroforestry, which combines rows of trees and crops, is a practice that is gaining increasing attention. The literature regarding this management strategy was examined by Buele et al. [11], showing that the inclusion of trees increases microbial abundance, community diversity, and metabolic activity compared to monocultures, which demonstrates the soil fertility benefits associated with agroforestry. Similarly, intercropping aims to exploit natural interactions among plants and their associated microbial species to enhance productivity and disease resistance. Li et al. [12] examined the effects of intercropping alfalfa with mulberry on the microbial community structures and physicochemical characteristics of soil, providing a framework for the optimal management of mulberry–alfalfa intercropping. While weeds are normally considered to be a nuisance in conventional monocropping agricultural production systems, Trinchera et al. [13] ask us to consider an alternative perspective in summarizing the literature on the arbuscular mycorrhizal fungi associated with weedy species. Their “agroecological” vision recognizes the role of naturally occurring plants in enhancing plant and soil microbial diversity. Tillage and residue management are common practices in agriculture, and Guan et al. [14] investigated the effects of these practices on soil microbial communities. Their results demonstrate that bacterial and fungal co-association networks respond differently to tillage, and that residue management plays a lesser role in shaping soil microbial communities. Biocontrol aims to optimize antagonistic microbe–microbe and beneficial microbe–plant interactions to provide natural pest control options for producers. Dong et al. [15] examined the genome of a root-associated plant growth-promoting bacterium, *Bacillus velezensis* SC60, demonstrating the presence of gene clusters encoding novel antimicrobials that inhibit pathogen growth and support root colonization by inhibiting other *Bacillus* spp. Tissues other than roots can also harbor beneficial bacteria. Gorrasi et al. [16] examined tomato peel epiphytes in plants grown under conventional and organic farming practices, demonstrating the potential to manipulate the presence of beneficial or harmful bacteria on the surfaces of fruit that is often consumed raw. Similarly, apple blossoms were shown to harbor microorganisms such as *Pantoea agglomerans* and *B. velezensis* that strongly inhibited the growth of the fire blight pathogen (*Erwinia amylovora*) [17]. In addition to antagonizing pathogen growth, bacteria associated with consumed fruit also affect fruit quality and aroma, and the mode of cultivation (conventional or organic) strongly affects the fruit-associated bacterial community structures [18]. The delivery of biocontrol microorganisms in a manner that will maximize their impact is of critical importance. Pereira et al. [19] review the literature on the use of biopolymeric matrices for this purpose, identifying a range of useful matrices such as alginate, starch, chitosan, and gelatin and underscoring the importance of future scale-up studies for commercial application.

Many microbe–host interactions in agricultural and forest ecosystems are harmful to the plant host. For example, vine decline caused by *Phytopythium* spp. has become an increasing impediment to kiwifruit production in Italy, and knowledge of the taxonomic range of pathogen species is important for disease management. Prencipe et al. [20] provide an inventory of the *Phytopythium* spp. found in Italian kiwi orchards, and suggest that their increasing presence may be supported by climatic warming currently being experienced. Nematodes are an often-neglected component of soil ecosystems, and two papers examine their role in shaping the composition and health of forest ecosystems. Pine wilt disease is caused by the pinewood nematode (PWD) *Bursaphelenchus xylophilus* and causes host mortality in pine forests. Liu et al. [21] examine the effect of PWD infection on the host microbiome, demonstrating that infection strongly affects the needle fungal community, with lesser effects on roots and soil communities. The critical role of nematodes in shaping forest soil microbial communities was examined using deadwood, which harbors a range of micro- and macro-biota whose activities result in carbon cycling and forest soil generation. Nematodes feeding on the bacteria and fungi that perform these roles, therefore, play a crucial role in establishing the ecological successions that occur during this process. Moll et al. [22] examine the nematode communities in deadwood, identifying almost 250 nematode taxa representing nearly 30 Families, including those feeding on bacteria and fungi. Their findings demonstrate the complexity of the ecological interactions that result in the degradation of woody residues, which feature a mixture of cooperativity and competition but provide the substrate for the forest soil and ultimately release fixed carbon back into the atmosphere.

Insights gained by examining the microbial components of terrestrial ecosystems are strongly influenced by the method used to provide them. Methods that provide a complete inventory of the microbial community composition, with minimal distortion, will enable better conclusions to be drawn and provide more useful information to inform management practices. Data analysis methods must also facilitate the identification of taxa and informative abundance patterns based on DNA sequence reads. The identification of microbial indicators, taxa with abundances that are related to various parameters, can be a useful means of providing a microbial taxonomic “fingerprint” associated with particular outcomes. Behnke et al. [23] discuss the use of this approach to examine the effect of long-term crop rotations on soil communities in agricultural production systems, identifying bacterial, fungal, and archaeal indicator species that are associated with different rotations and nutrient compositions. The generation of DNA sequence data can result in analytical biases that should be minimized as much as possible. PCR amplification, a commonly used method for enriching environmental DNA for taxonomic markers, inevitably introduces both representational and abundance biases into the data, and the use of the 16S rRNA gene limits taxonomic identification below the Genus level. Links et al. [24] demonstrate the use of hybridization probes targeting a high-resolution taxonomic marker, chaperonin-60, to obviate these limitations. Finally, DNA sequence data are inherently compositional, providing only relative abundances of the taxa within a microbial ecosystem [25]. Beule et al. [26] address this limitation, demonstrating that such relative abundances can lead to inaccurate estimates of microbial populations and community dynamics. Their work, which examined fungal taxa in an agroforestry management system, emphasizes the importance of supplementing amplicon sequencing (providing relative abundances) with the absolute quantification of taxonomic abundances generated using methods such as qPCR.

The study of microbial life in natural and managed terrestrial ecological niches using culture-independent, DNA sequencing-based methods provides critical information on the composition and dynamics of these important ecosystems. While Kropotkin may have erred in selectively overemphasizing the importance of high cooperation/low conflict interactions in shaping biological responses to environmental conditions, his ideas nevertheless laid the foundation for the much more recent concept of the holobiont [27], the extension of the individual to include the host and its microbial symbionts. Further consideration of the full complexity of interactions within ecosystems is leading to a reconsideration of the nature of organismality itself [28,29]. Continued study of the invisible microbial majority using the most accurate, complete, and unbiased methods available will surely lead to further conceptual refinements that will enable the development and application of management methods supporting the health and productivity of the forest, agricultural, and agroforestry ecosystems.
